# Krüppel‐like factor 4 regulates stemness and mesenchymal properties of colorectal cancer stem cells through the TGF‐β1/Smad/snail pathway

**DOI:** 10.1111/jcmm.14882

**Published:** 2019-12-12

**Authors:** Zhengwei Leng, Yong Li, Guojun Zhou, Xiaojiang Lv, Walden Ai, Jianshui Li, Lingmi Hou

**Affiliations:** ^1^ Northeast Sichuan Acute Pancreatic Research Center North Sichuan Medical College Sichuan China; ^2^ Cancer Stem Cells Research Center Affiliated Hospital of North Sichuan Medical College Sichuan China; ^3^ Department of Biology, Chemistry and Environmental Health Science Benedict College Columbia SC USA; ^4^ Thyriod and Breast Surgery Affiliated Hospital of North Sichuan Medical College Sichuan China

**Keywords:** cancer stem cell, colorectal cancer, Krüppel‐like factor 4, snail, TGF‐β1

## Abstract

Krüppel‐like factor 4 (KLF4) was closely associated with epithelial‐mesenchymal transition and stemness in colorectal cancer stem cells (CSCs)‐enriched spheroid cells. Nonetheless, the underlying molecular mechanism is unclear. This study showed that KLF4 overexpression was accompanied with stemness and mesenchymal features in Lgr5^+^CD44^+^EpCAM^+^ colorectal CSCs. KLF4 knockdown suppressed stemness, mesenchymal features and activation of the TGF‐β1 pathway, whereas enforced KLF4 overexpression activated TGF‐β1, phosphorylation of Smad 2/3 and Snail expression, and restored stemness and mesenchymal phenotypes. Furthermore, TGF‐β1 pathway inhibition invalidated KLF4‐facilitated stemness and mesenchymal features without affecting KLF4 expression. The data from the current study are the first to demonstrate that KLF4 maintains stemness and mesenchymal properties through the TGF‐β1/Smad/Snail pathway in Lgr5^+^CD44^+^EpCAM^+^ colorectal CSCs.

## 1 INTRODUCTION

Cancer metastasis contributes to approximately 90% of cancer‐related deaths.^1^ Accumulating evidence indicates that cancer stem cells (CSCs) are responsible for the seeding and colonization of cancer metastasis.^2^, ^3^, ^4^ For the past decades, studies have explored and investigated the mechanisms of human carcinogenesis to help develop novel therapeutic strategies. To date, cancer treatment has not been much improved in advanced and chemo‐resistant human cancers. Thus, a better understanding of CSCs behaviour in human cancers, including colorectal cancers (CRC), could provide insight into defining the molecular mechanisms of tumorigenesis and cancer progression. Indeed, identification of CSC markers is a very hot topic and interesting data have been generated.^5^ For example, CSC markers for breast, prostate, colon and pancreatic cancers have been reported.^6^ Further evidence has suggested the association of CSC phenotypes (stemness) and epithelial‐mesenchymal transition (EMT) progress, of which the epithelial makers E‐cadherin (E‐cad) and ZO‐1 are down‐regulated, whereas the mesenchymal markers N‐cadherin (N‐cad), Vimentin (Vim), Snail and Slug are up‐regulated.^7^ After acquiring mesenchymal properties, cancer cells can invade other organs by forming metastases.^7^ However, the way in which stemness and mesenchymal properties are connected, as well as its relevance for the metastatic process, are still unknown.

Recent studies have shown that TGF‐β1 related to cancer malignant behaviours.^8^, ^9^ For example, in CRC, TGF‐β1 highly expression promoted cancer progression by Smad signalling. Moreover, TGF‐β1 signalling regulates EMT‐related genes, thereby facilitating cancer progression in CRC.^10^, ^11^ Towards this end, our research has focused on Krüppel‐like factor 4 (KLF4), a transcription factor that was involved in regulation of cell proliferation, apoptosis, differentiation and cancer progression.^12^, ^13^ In CRC cells, we found that KLF4 acted as an oncogene in colorectal CSCs‐enriched spheroid cells.^14^ However, in a CSCs model, it needs further investigation of the underlying mechanisms of KLF4, stemness and EMT.

## 2 MATERIALS AND METHODS

### 2.1 Ethics statement

The informed written consent was obtained from patients. This study was conducted in accordance with the Declaration of Helsinki and approved by the institution's ethics board (Permit Number#S255).

### 2.2 Cell lines and culture

Human CRC cell lines DLD‐1, HCT116 and HT29 were obtained from the American Type Culture Collection (ATCC) and authenticated by short tandem repeat PCR profiling at ATCC. The cell lines were obtained in March 2016 and immediately cultured for experiments. The medium used to culture each cell line was according to the ATCC, and cells were cultured in a humidified incubator with 5% CO_2_ at 37°C.^14^

### 2.3 Primary cancer tissue samples and tumour cell isolation

The patients were histologically diagnosed with CRC and surgically treated in our hospital (Table S1). During the surgery, fresh tumour tissues were resected and stored in phosphate‐buffered saline (PBS) containing 1000 unit/mL penicillin, 1000 μg/mL streptomycin and 3 μg/mL fungizone. After transportation to the research laboratory, the tissue specimens were chopped into 1 × 1 mm^3^ pieces and digested with 2 mg/mL of collagenase (Invitrogen) for 2 hours at 37°C on a shaker. Next, cells were dispersed by passing through a pipette tip and filtrating through a 100 and 53 μm nylon cell strainer. The cells were then incubated in PBS containing 1000 units/mL penicillin, 1000 μg/mL streptomycin and 3 μg/mL fungizone at 37°C for 30 minutes. Primary human cancer cells were collected and made of a single cell suspension culture by adding serum‐free media (see below).

### 2.4 Flow cytometry

Immunostaining of proteins was conducted as described in previous studies with some modifications.^15^, ^16^ In brief, cells were fixed and permeabilized with BD Pharmingen Transcription Factor Buffer Set (BD Bioscience) for 40 minutes at the room temperate according to the manufacturer's instructions. The cells were then incubated for 40 minutes with a 1/100 dilution of primary antibodies, or polyclonal rabbit immunoglobulins (Cell Signaling Technology) as the isotypic control. Cells were then washed with PBS and further incubated with 1/100 dilution of FITC‐/or APC‐conjugated goat anti‐rabbit IgG (R&D) for 30 minutes at 37°C. Samples were washed with PBS and resuspended in 500 μL of PBS for flow cytometric analysis using a BD FACSVerse flow cytometer (BD Bioscience). The data were then quantified with the FlowJo software, version 10.0 (BD Bioscience). We analysed the protein expression using ratio of fluorescence intensity (RFI), which was calculate by the mean fluorescence intensity of the stained samples to the isotypic control samples. Cells were delegated as positive if the RFI > 2 (Gaussian distribution). The primary antibodies used were an anti‐KLF4 (Santa Cruz Biotechnology), E‐cad, Vim, Snail, Slug, ZO‐1, Smad2, Smad3, p‐Smad2, p‐Smad3, Smad4 and TGF‐β1 antibodies (Cell Signaling Technology).

### 2.5 Lgr5^+^CD44^+^EpCAM^+^ cells culture and infection

Human Lgr5^+^CD44^+^EpCAM^+^ cells were sorted and cultured according to our previous study^17^ Lentivirus carrying KLF4 cDNA or KLF4 shRNA was purchased from Shanghai GeneChem Co., Ltd. The stably transfected cells were generated according to previous studies^14^, ^18^ and then treated with 10 μg/mL puromycin for 48 hours. To block the TGF‐β1 pathway, we treated cells with 2 μg/mL SB525334, a TGF‐β1 inhibitor for 6 hours.^19^

### 2.6 RNA isolation and qRT‐PCR

Total cellular RNA was isolated using TRIzol Reagent (Invitrogen), and mRNA levels were measured by qRT‐PCR as described previously.^14^ The primer pairs for measuring the levels of each mRNA are listed in Table S2.

### 2.7 Sphere‐forming assay

CSCS (1 × 10^3^ cells per well) were plated in six‐well plates and cultured in serum‐free DMEM/F12 medium were conducted as described previously.^17^ The number of spheres per well was counted.

### 2.8 Limiting dilution assay

Limiting dilution assay (LDA) and tumour sphere‐forming assay were conducted as described previously with some modifications to calculate the percentages of CSCs.^20^

### 2.9 Colony formation assay

Colony formation assay was performed as described previously.^14^ Briefly, CSCs (1 × 10^3^ cells per well) were plated into six‐well plates and cultured for two weeks. After stained with violet, cells were photographed and accounted for their in vitro tumorigenic efficiency.

### 2.10 Immunofluorescence staining and laser confocal microscopy

CSCs were sorted and grown in serum‐free DMEM/F12 medium in glass‐coverslips for 16 hours before fixed with 2% paraformaldehyde at 37°C for 15 minutes. The fixed cells were then permeabilized with 0.1% Triton X‐100 at the room temperature for 15 minutes followed by incubation at 37°C for 2 hours with primary antibody against KLF4 (Santa Cruz Biotechnology) or TGF‐β1 (Cell Signaling Technology). Cells were then incubated with FITC‐conjugated or APC‐conjugated secondary antibodies (Santa Cruz Biotechnology) at the room temperature for 1 hour and mounted with DAPI (Invitrogen). Images were taken using Olympus FV1200 microscope.

### 2.11 Real‐time tumour cell migration and invasion assay

The migration and invasion capacities were detected by xCELLigence system (RTCA DP Station) as previously described.^21^ This system provides a real‐time detection of migration and invasion of cells by extrapolating from the data in electrical impedance with the number of cells passing through a porous membrane. Briefly, 150 μL of RPMI1640 complete cell culture medium was added on the lower chamber plate (Roche Diagnostics Co.). The upper chamber was coated with (for invasion) or without (for migration) Matrigel (Sigma Chemicals). After 50 minutes of equilibration using RPMI1640 medium, 4 × 10^4^ cells were plated onto the upper chamber. The plates were subsequently placed on the RTCA analyser in incubator. The migration and invasion data were recorded every 20 minutes. In addition, the traditional Transwell migration and invasion assay were performed as described previously.^14^

### 2.12 In vivo model of tumour cell xenograft assay

Single cells (1 × 10^5^ cells) were subcutaneously injected into 4‐ to 6‐week‐old female C57BL/6 nude mice with a body weight of 25.2 ± 2.13 g (n = 5 per group; Beijing HFK Bioscience). Mice were housed in a specific pathogen‐free, environmentally controlled facility with food and water available ad libitum. The housing was maintained at constant room temperature (21 ± 2°C) and humidity (45%), and kept under a regular 12‐hour light/dark schedule with lights on from 08:00 am to 20:00 pm. Mice were then sacrificed with pentobarbital sodium (100 mg/kg, Sigma‐Aldrich or Merck) intraperitoneal injection when grafts reached a length of 2.0 cm, or 60 days after injection, whenever it came first.^14^ Tumours were then histopathological analysed. Tumour volume was calculated using length × width^2^ × 0.5. This study was approved by the Institutional Animal Care and Use Committee (IACUC) of Huazhong University of Science and Technology.

### 2.13 Histologic analysis

Mouse tumour xenografts were fixed in formalin and then embedded in paraffin. Tumour tissue sections were prepared and stained with H&E. Images were then taken with an Olympus IX71 (Olympus).

### 2.14 Statistical analysis

Statistical analyses were conducted using the SPSS software, version 19.0 (SPSS). All data were expressed as the mean ± SD. Student's *t* test and one‐way ANOVA were used to evaluate the significant associations among categorical variables. Data with a value of *P* < .05 were considered statistically significant.

## 3 RESULTS

### 3.1 Expression of KLF4 in Lgr5^+^CD44^+^EpCAM^+^ colorectal CSCs

Our previous study demonstrated that colorectal CSCs were highly restricted to Lgr5^+^ subpopulations. Moreover, Lgr5 combined with CD44 and EpCAM might assist make strides the stem‐like characteristics of colorectal CSCs.^17^ To delineate the Lgr5^+^CD44^+^EpCAM^+^ cells in CRC, we measured the percentage of Lgr5^+^CD44^+^EpCAM^+^ cells in various human CRC cell lines and tissue samples using flow cytometry (Table S3). We found that DLD‐1 cells had the highest percentages of Lgr5^+^CD44^+^EpCAM^+^ cells. Therefore, Lgr5^+^CD44^+^EpCAM^+^ cells from DLD‐1, and seven tissue samples (patient #1, 3, 4, 6, 8, 11, 12) sorted by flow cytometry were used for further study.

Our data showed that the level of KLF4 expression was significantly higher in Lgr5^+^CD44^+^EpCAM^+^ cells than those of Lgr5^−^CD44^−^EpCAM^−^ cells (Figure S1A). The Lgr5^+^CD44^+^EpCAM^+^ cells also expressed high levels of transcripts of stem cells and CSC genes, such as Oct4, Sox2, Nanog, CD133, CD44 and TGF‐β1 (Figure S1A). Moreover, mesenchymal genes, such as N‐cad, Vim, Snail and Slug, were highly expressed in Lgr5^+^CD44^+^EpCAM^+^ cells compared with Lgr5^−^CD44^−^EpCAM^−^ cells, whereas the epithelial markers ZO‐1 and E‐cad were overexpressed in Lgr5^−^CD44^−^EpCAM^−^ cells (Figure S1A). We measured the co‐expression of TGF‐β1 and KLF4 in the same cells by immunofluorescence staining and laser confocal scanning (Figure S1B). More importantly, Lgr5^+^CD44^+^EpCAM^+^ cells had the capacity to form spheres when passaged in sphere‐forming conditions for multiple generations, indicating self‐renewal capabilities (Figure S1C). These data indicated that KLF4 expression was associated with stemness, mesenchymal properties and TGF‐β1 expression in human colorectal CSCs.

### 3.2 KLF4 overexpression facilitates colorectal CSCs stemness properties

To further confirm that KLF4 was indispensable in maintaining the stemness and mesenchymal phenotypes in colorectal CSCs, we conducted gene knockdown and overexpression experiments by generated stable KLF4 knockdown Lgr5^+^CD44^+^EpCAM^+^ cells (designated as CSCs‐shKLF4) and KLF4 overexpression Lgr5^+^CD44^+^EpCAM^+^ cells (designated as CSCs‐KLF4) according to a previous study, while control cells were designated as CSCs‐shCon.^14^

**Figure 1:**
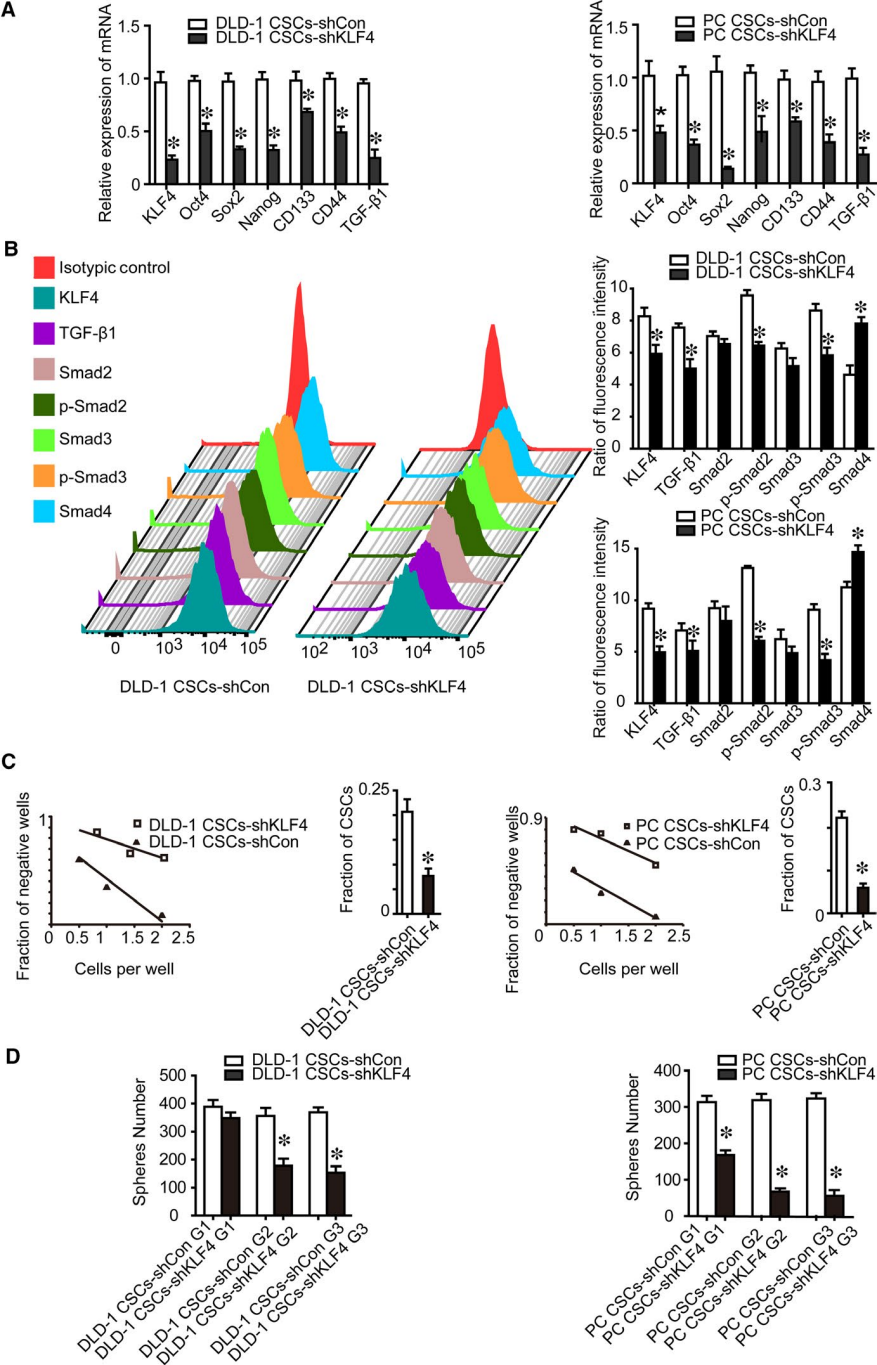
Effect of KLF4 knockdown on the stemness properties of Lgr5^+^CD44^+^EpCAM^+^ cells and expression of the TGF‐β1 pathway key genes. A, KLF4 knockdown resulted in decreased expression of stem cell core gene Oct4, Sox2 and Nanog, and cancer stem cells gene CD133, CD44 and TGF‐β1 detected by using qRT‐PCR. B, KLF4 knockdown resulted in decreased expression of TGF‐β1, p‐Smad2, p‐Smad3 proteins, while increased expression Smad4 protein detected by using flow cytometry. C, The number of cancer stem cells decreased after KLF4 knockdown detected by using the limiting dilution assay. D, The capacity of self‐renewal decreased after KLF4 knockdown as detected by sphere‐forming assay. G1, Generation 1; G2, Generation 2; G3, Generation 3; the data represented as mean ± SD of three replicated experiments (*P < .05)

We found that knockdown of KLF4 expression was associated with a significant decrease in transcripts of stem cell and CSC‐related genes (Figure 1A). Moreover, KLF4 knockdown down‐regulated TGF‐β1, p‐Smad2 and p‐Smad3. Conversely, Smad4, a well‐known tumour silencer and a major regulator of intracellular TGF‐β1 signalling, was up‐regulated after knockdown of KLF4 expression (Figure 1A,B).^22^ Knockdown of KLF4 expression also strongly reduced the number of CSCs as assessed by a LDA (Figure 1C). Because a sphere is composed of all descendants from a single CSC, the number of sphere reflects the CSC population^23^ and CSC frequency can be estimated through the LDA.^20^, ^24^, ^25^ Our data showed that the median frequencies were from 100/211 of CSCs‐shCon cells to 100/566 of CSCs‐shKLF4 cells in primary colorectal patient samples, and the median frequencies were decreased in Lgr5^+^CD44^+^EpCAM^+^ cells from DLD‐1 (100/484 vs 100/1304) cells after KLF4 knockdown (Figure 1C). These data are consistent with an obligate role for KLF4 in maintaining stemness in colorectal CSCs.

**Figure 2:**
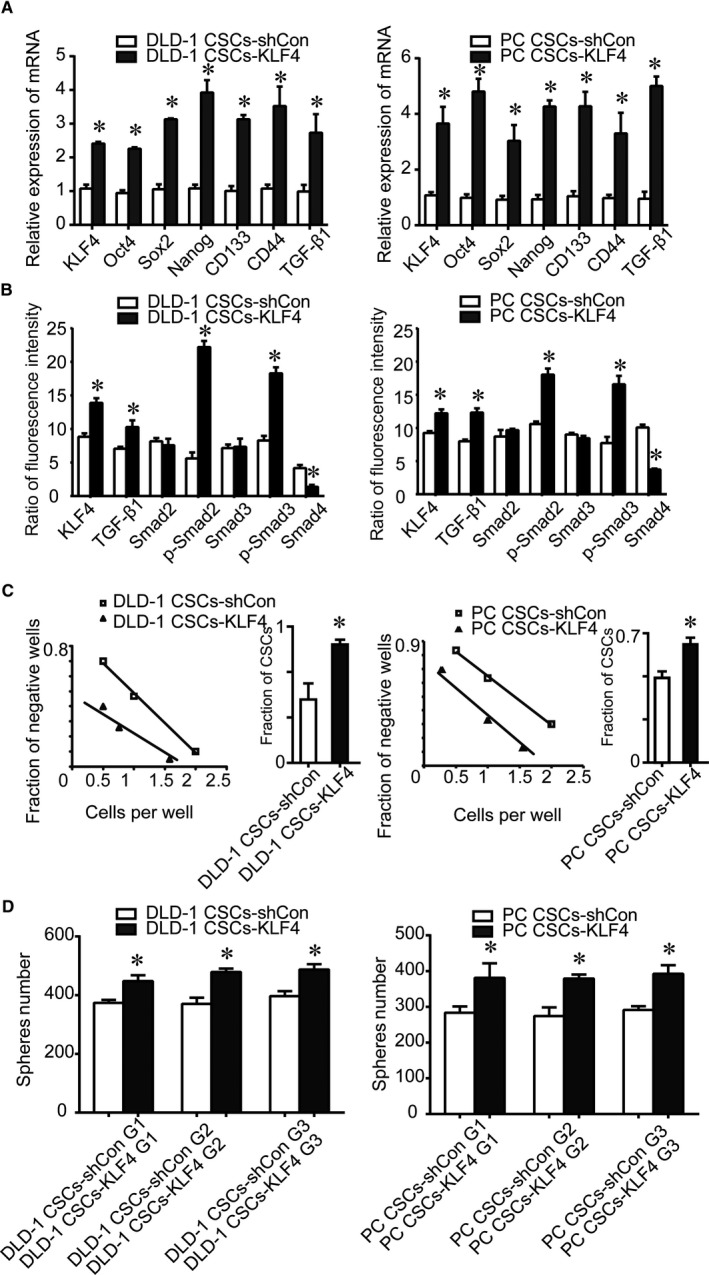
Effect of KLF4 overexpression on the stemness properties of Lgr5^+^CD44^+^EpCAM^+^ cells and expression of the TGF‐β1 pathway key genes. A, mRNA expression of stem cell core genes Oct4, Sox2 and Nanog, and cancer stem cells genes CD133, CD44 and TGF‐β1 increased after KLF4 overexpression detected by using qRT‐PCR. B, KLF4 overexpression increased expression of TGF‐β1, p‐Smad2, p‐Smad3 proteins, and decreased expression of Smad4 protein as detected by using flow cytometry. C, The number of cancer stem cells increased after KLF4 overexpression as detected using the limiting dilution assay. D, The capacity of self‐renewal increased after KLF4 overexpression as detected by sphere‐forming assays. The data represented as mean ± SD of three replicated experiments (*P < .05)

To determine whether KLF4 plays a role in CSC self‐renewal, we performed serial sphere‐forming assays and found that there were significantly fewer shKLF4 multipotent spheres than shCon spheroid cells, indicating a decrease in shKLF4 cell self‐renewal. Moreover, shKLF4 spheres were significantly smaller than shCon cell spheres, suggesting a decreased CSC proliferative ability in the shKLF4 spheroid culture. Remarkably, knockdown of KLF4 expression prevented the formation of second and third‐generation shKLF4 spheres, whereas we observed the formation of secondary and third generations of shCon spheres (Figure 1D). Furthermore, KLF4 overexpression in Lgr5^+^CD44^+^EpCAM^+^ cells (CSCs‐KLF4) increased the number of CSCs, self‐renewal capacity, and stem cell and CSC‐related genes expression (Figure 2). KLF4 overexpression up‐regulated active TGF‐β1, active p‐Smad2/3, but down‐regulated Smad4 (Figure 2). Collectively, these results demonstrated that KLF4 overexpression confers stemness properties in human colorectal CSCs.

### 3.3 KLF4 overexpression facilitates colorectal CSCs mesenchymal phenotypes

**Figure 3:**
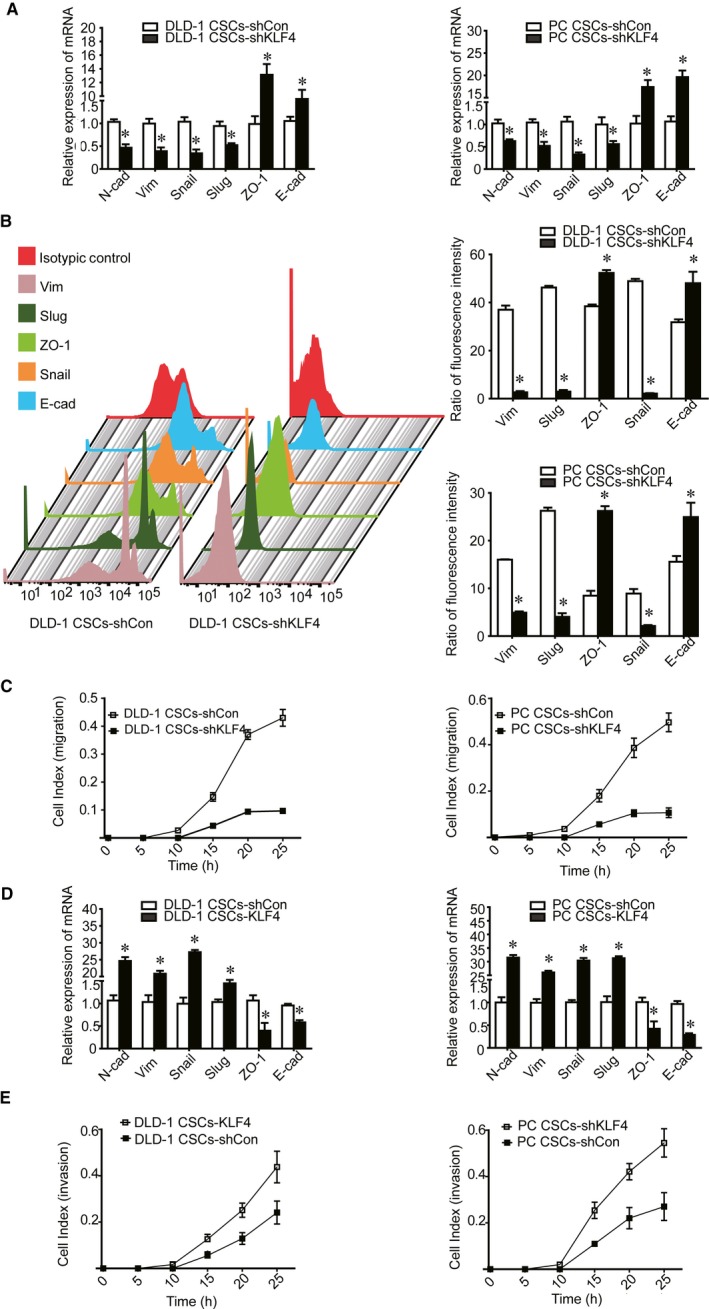
Effect of KLF4 on the mesenchymal properties of Lgr5^+^CD44^+^EpCAM^+^ cells. A‐B, KLF4 knockdown resulted in decreased expression of N‐cad, Vim, Snail, Slug, but increased expression of E‐cad and ZO‐1 mRNA and protein. C, Tumour cell migration and invasion capabilities were suppressed after KLF4 knockdown as detected by real‐time migration and invasion assay. D, KLF4 overexpression increased N‐cad, Vim, Snail, Slug expression, and decreased expression of E‐cad and ZO‐1. E, Tumour cell migration and invasion capabilities increased after KLF4 overexpression as detected by using the real‐time migration and invasion assay. The data represented as mean ± SD of three replicated experiments (*P < .05)

We next determined whether KLF4 expression could affect CSCs EMT. Our data showed that knockdown of KLF4 expression was associated with decreased expression of N‐cad, Vim, Snail and Slug, but increased ZO‐1 and E‐cad expression (Figure 3AB). In addition, knockdown of KLF4 expression suppressed migration and invasion of CSCs as determined by real‐time tumour cell migration and invasion assays (Figure 3C).

KLF4 overexpression in CSCs also increased N‐cad, Vim, Snail and Slug expression, but decreased ZO‐1 and E‐cad expression (Figure 3D). Moreover, KLF4 overexpression restored CSCs migration and invasion capacity (Figure 3E). These data suggested KLF4 overexpression promoted the mesenchymal phenotypes in Lgr5^+^CD44^+^EpCAM^+^ cells.

### 3.4 KLF4 regulates CSCs stemness and mesenchymal properties through the TGF‐β1/snail pathway

Our data showed that KLF4 overexpression could promote CSC EMT and TGF‐β1 pathway activation. A previous study demonstrated that TGF‐β1 serves as a strong driver to promote cancer progression by facilitating EMT.^26^ Upon incitement by TGF‐β1, Smad2/3 is enacted after phosphorylation and after that related with Smad4 to in turn translocate into the nucleus to activate transcription of EMT genes, such as Snail.^27^ As a crucial EMT inducer, Snail acts as an upstream factor that can induce multiple EMT transcription factors, like Slug, Zeb1 and Twist1.^28^ Exogenous overexpression of Snail reportedly increases the invasive and metastatic abilities of cancer cells by the induction of EMT.^29^ Snail is known to promote aggressive cancer cell phenotypes, even though its regulatory role in CSC malignant profiles remains unknown.^29^, ^30^, ^31^ Therefore, we determined whether KLF4 maintains the stemness and mesenchymal phenotypes through the TGF‐β1/Smad/Snail‐dependent signalling. We first confirmed the co‐expression of KLF4 and TGF‐β1 by immunofluorescence staining and, as expected, KLF4 and TGF‐β1 proteins were co‐expressed in the nucleus of colorectal Lgr5^+^CD44^+^EpCAM^+^ cells (Figure S1B). We then treated KLF4 overexpression CSCs with TGF‐β1 inhibitor SB525334 and assessed stemness and mesenchymal properties.^19^

**Figure 4:**
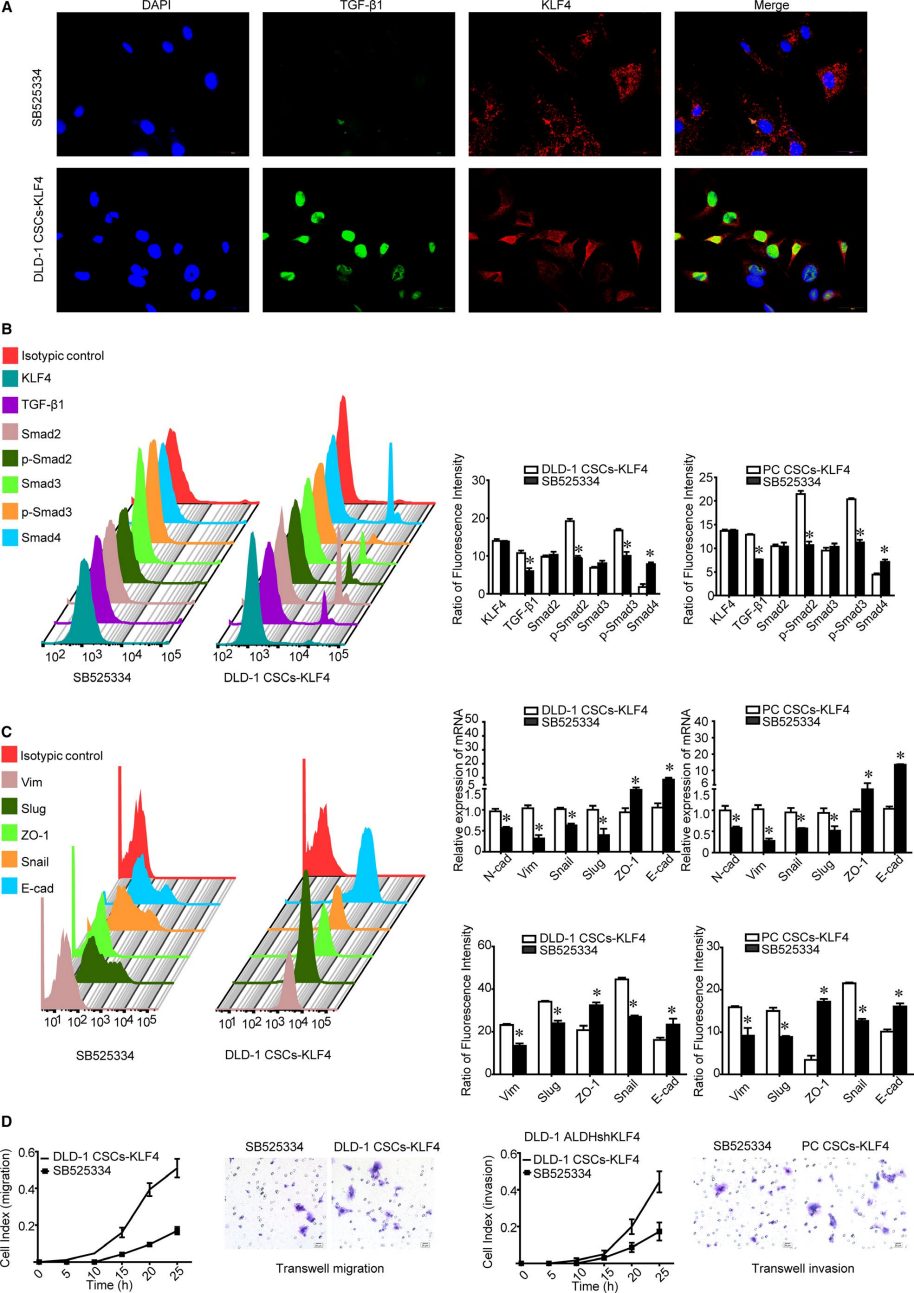
Effect of SB525334 on KLF4 overexpression‐induced mesenchymal phenotypes of Lgr5^+^CD44^+^EpCAM^+^ cells. A, SB525334 decreased expression of TGF‐β1 detected by using immunofluorescence staining and laser confocal microscopy. Scale bars, 30 μm. B, SB525334 decreased the expression of TGF‐β1, p‐Smad2, p‐Smad3 protein and increased Smad4 protein. C, SB525334 decreased expression of N‐cad, Vim, Snail, Slug proteins, and increased expression of E‐cad and ZO‐1 proteins. D, SB525334 decreased the migration and invasion capabilities of KLF4 overexpressed CSCs detected by using the real‐time migration and invasion assay and Transwell migration and invasion assay, respectively. Scale bars = 20 μm. The data represented as mean ± SD of three replicated experiments (*P < .05)

As anticipated, SB525334 down‐regulated TGF‐β1 and p‐Smad2/3 expression, but up‐regulated Smad4 in KLF4‐overexpressing Lgr5^+^CD44^+^EpCAM^+^ cells (Figure 4AB). Because the TGF‐β1 signalling activation causes tumour cell EMT and metastasis, we investigated the impact of SB525334 on CSCs EMT and stemness and observed a decrease in mesenchymal protein expression and an increase in the epithelial marker ZO‐1 and E‐cad expression (Figure 4C). Snail, which can bind to the E‐cad promoter to repress E‐cad transcription, was also down‐regulated. However, KLF4 expression was unchanged. In addition, SB525334 abolished KLF4‐facilitated migration and invasion of CSCs (Figure 4D).

**Figure 5:**
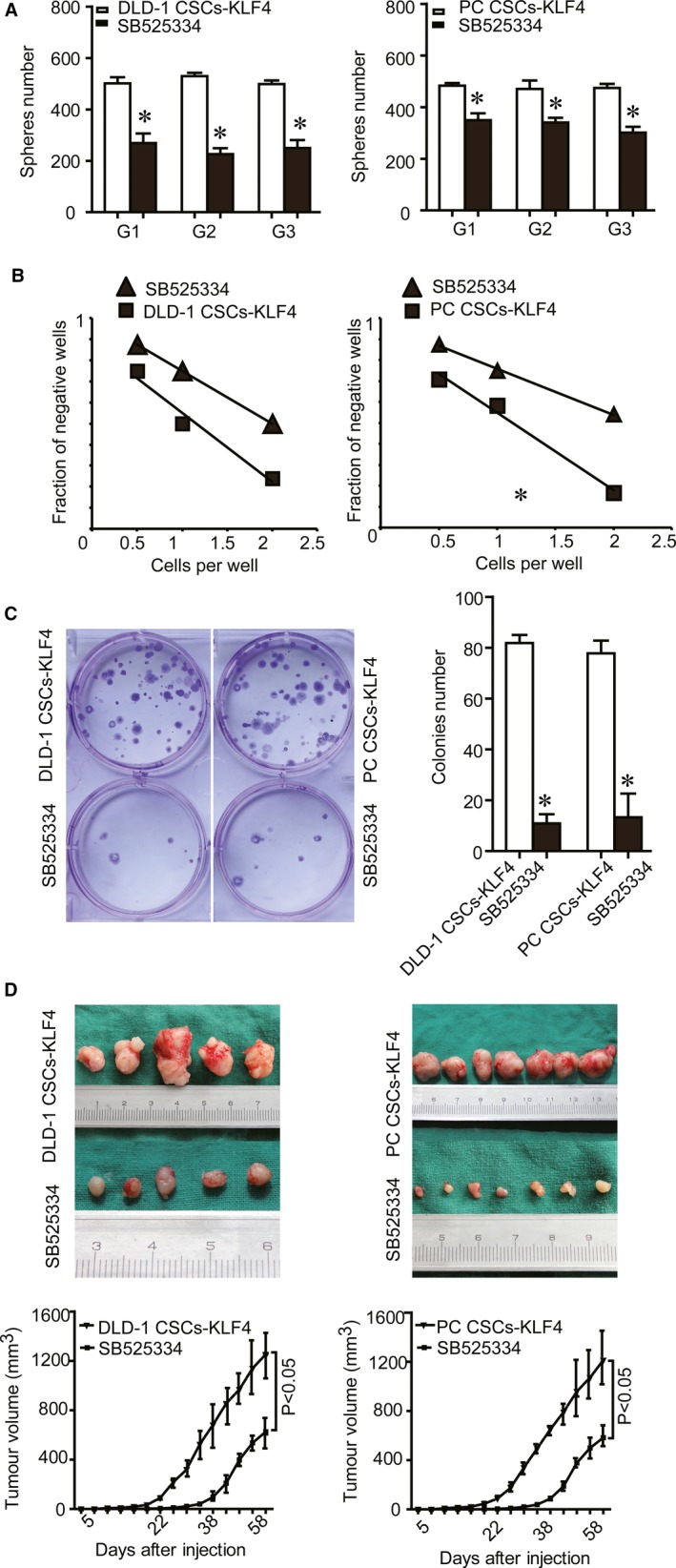
Effect of SB525334 on KLF4 overexpression‐induced stemness and tumorigenesis of Lgr5^+^CD44^+^EpCAM^+^ cells. A, The capacity of self‐renewal decreased after SB525334 treatment as detected using the sphere‐forming assay. B, The number of cancer stem cells decreased after SB525334 treatment as detected by using the limiting dilution assay. C, The number of colonies decreased after SB525334 treatment as detected by the colony formation assay. D, Tumorigenesis in vivo was suppressed after SB525334 treatment as detected by using the tumour cell xenograft formation assay. The data were represented as mean ± SD of three replicated experiments (*P < .05)

SB525334 also decreased the stemness properties of CSCs, such as self‐renewal, percentage of CSCs as assessed by sphere formation assay and LDA, respectively (Figure 5A,B). Finally, we demonstrated that SB525334 abrogated the effect of KLF4 overexpression on the tumorigenic properties of CSCs in vivo and in vitro (Figure 5CD). Collectively, KLF4 promotes stemness and mesenchymal phenotypes in colorectal CSCs through the TGF‐β1/Smad/Snail signalling.

## 4 DISCUSSION

Emerging evidence strongly supports CSC existence in various solid tumours and these CSCs or tumour‐initiating cells are responsible for initiating and sustaining tumour development and progression.^2^, ^3^ Therefore, CSCs have recently gained increasing attention in the field of cancer research. Experimental systems that isolate, quantify and functionally characterize CSCs are critical to study the pathways included in self‐renewal and recognize CSC‐specific therapeutic targets.^32^, ^33^, ^34^ Human colorectal CSCs have been well recognized by their specific markers, such as CD133, CD44, CD24, EpCAM, ALDH and Lgr5 together with the CSC malignant profiles, including spheroid and colony formation.^14^, ^35^, ^36^ Our previous study indicated that Lgr5^+^CD44^+^EpCAM^+^ strictly defined the CSC lineage in human CRC.^17^ Other studies demonstrated an association of the CSC phenotype with mesenchymal properties, and CSCs were reported to acquire mesenchymal characteristics and promote their ability of self‐renewal, invasion and metastasis.^37^, ^38^ However, the underlying molecular mechanisms between stemness and mesenchymal properties remain to be defined. In the current study, we investigated whether KLF4 exerts its oncogenic functions by maintaining stemness and mesenchymal properties in CSCs because our previous studies demonstrated that KLF4 acted as an oncogene in human colorectal CSC‐enriched spheroid cells.^14^, ^39^ We found for the first time that KLF4 is required to maintain the stemness and mesenchymal properties through the TGF‐β1/Smad/snail pathway in human colorectal Lgr5^+^CD44^+^EpCAM^+^ CSCs.

TGF‐β1 highly expression within the cancer microenvironment restrains immune surveillance therefore promotes the cancer invasion and enhances anti‐cancer immune responses resistance.^40^ Previous studies showed that TGF‐β1 stimulation caused phosphorylation of Smad2 and Smad3 and in turn their accumulation in the nucleus to promote Snail transcription,^41^ which in turn regulates EMT by effects on epithelial cell markers such as E‐cad.^42^

To further study the mechanism of KLF4 maintenance on stemness and mesenchymal phenotypes in CSCs, we knocked down and overexpressed KLF4 in cells and found that knockdown of KLF4 expression decreased stemness properties, along with decreased TGF‐β1, p‐Smad2/3 and up‐regulated Smad4.^43^, ^44^ Moreover, KLF4 knocking‐down effectively suppressed tumour cell migration and invasion capability, along with a decrease in expression of mesenchymal genes, such as Snail, a crucial downstream activator of TGF‐β1/Smad pathway.^45^ However, KLF4 overexpression also restored stemness and mesenchymal phenotypes. Meanwhile, KLF4 overexpression induced TGF‐β1 expression consequently activated p‐Smad2/3. On the contrary, Smad4, a cancer suppressor and an important mediator of TGF‐β1 pathway, was down‐regulated by KLF4 overexpression. These data support the obligate role of KLF4 in maintaining stemness and mesenchymal properties of human colorectal CSCs through the TGF‐β1/Smad/Snail pathway.

Snail was reported as an upstream factor of three different EMT‐inducing protein families, namely the Snail (including Snail and Slug), ZEB (including ZEB1 and ZEB2) and basic helix‐loop‐helix (including TWIST1, TWIST2 and TCF3) families.^28^, ^46^ Although several studies have shown that KLF4 negatively regulated the Snail family and EMT,^47^, ^48^ we asked whether KLF4 maintains mesenchymal properties by the TGF‐β1/Smad/Snail activation. In the CSC research models, our current study, for the first time, showed that expression of TGF‐β1, p‐Smad2/3 and Snail was down‐regulated after KLF4 knockdown, whereas KLF4 overexpression enhanced expression of TGF‐β1, p‐Smad2/3, and Snail and restored CSC stemness and mesenchymal phenotypes. Moreover, a TGF‐β1 inhibitor SB525334 abolished KLF4‐induced EMT and stemness without affecting KLF4 expression. As shown in Figure 4, SB525334 reduced expression of TGF‐β1, p‐Smad2/3 and Snail, but induced expression of Smad4, E‐cad and ZO‐1 relative to KLF4 overexpression Lgr5^+^CD44^+^EpCAM^+^ CSCs. Meanwhile, SB525334 abolished KLF4 overexpression and induced tumorigenesis in vitro and in vivo.

In summary, our current study for the first time identified that KLF4 was able to maintain the stemness and mesenchymal properties in human colorectal CSC models through the TGF‐β1/Smad/Snail pathway. Thus, KLF4/TGF‐β1/Smad/Snail is an attractive strategy for the treatment of CRC s specifically by targeting CSCs.

## CONFLICT OF INTEREST

The authors confirm that there are no conflicts of interest.

## AUTHOR CONTRIBUTION

Conceived and designed the experiments: Leng Zhengwei. Performed the experiments: Leng Zhengwei, Li Yong, Zhou Guojun, Lv Xiaojiang. Analysed the data: Walden Ai, Li Jianshui, Hou Lingmi. Contributed reagents/materials/analysis tools: Walden Ai, Li Jianshui, Hou Lingmi. Wrote the paper: Leng Zhengwei.

## Data Availability

The data that support the findings of this study are available from the corresponding author upon reasonable request.
