# Supplementary figures and images for: Krüppel‐like factor 4 regulates stemness and mesenchymal properties of colorectal cancer stem cells through the TGF‐β1/Smad/snail pathway
Source: J Cell Mol Med. 2019 Dec 12;24(2):1866–77. doi: 10.1111/jcmm.14882 (PMC6991673; doi:10.1111/jcmm.14882)

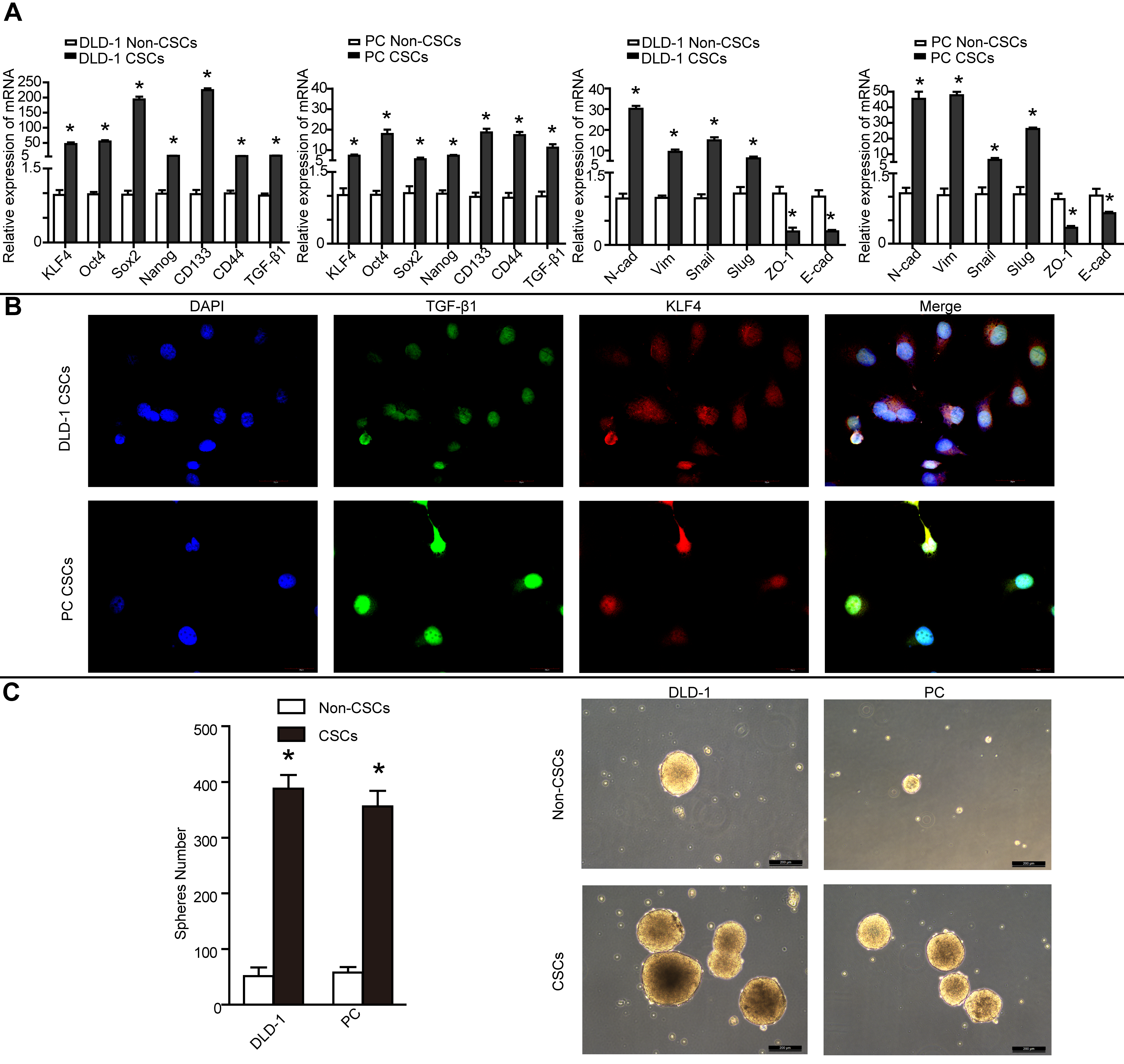

Supplement: Supplementary file 1 [file JCMM-24-1866-s001.tif]
