# Supplementary material for: Krüppel‐like factor 4 regulates stemness and mesenchymal properties of colorectal cancer stem cells through the TGF‐β1/Smad/snail pathway
Source: J Cell Mol Med. 2019 Dec 12;24(2):1866–77. doi: 10.1111/jcmm.14882 (PMC6991673; doi:10.1111/jcmm.14882)
Supplement: Supplementary file 2 [file JCMM-24-1866-s002.docx]

**Table S1.** Clinicopathological characteristics of colorectal cancer patients.

| **Case #** | **Gender** | **Age** | **Differentiation** | **Tumor stage** | **Size (cm)** |
| --- | --- | --- | --- | --- | --- |
| patients #1 | male | 49 | middle | T_4_N_2_M_0_ | 4.8*3.8 |
| patients #2 | female | 58 | well-middle | T_4_N_2_M_0_ | 3*3 |
| patients #3 | male | 51 | middle | T_2_N_1_M_0_ | 2*1.5 |
| patients #4 | male | 34 | middle | T_4_N_0_M_0_ | 4.5*3 |
| patients #5 | female | 60 | well-middle | T_4_N_1_M_0_ | 3.2*2.1 |
| patients #6 | male | 55 | middle | T_4_N_0_M_0_ | 3.5*3 |
| patients #7 | male | 40 | middle | T_4_N_2_M_0_ | 3.2*2.8 |
| patients #8 | female | 48 | middle | T_3_N_2_M_0_ | 6*1.5 |
| patients #9 | male | 58 | middle | T_2_N_2_M_0_ | 3.7*2.6 |
| patients #10 | female | 61 | well-middle | T_4_N_2_M_0_ | 3.3*1.7 |
| patients #11 | male | 50 | well-middle | T_4_N_2_M_0_ | 4.6*3.3 |
| patients #12 | female | 69 | middle | T_4_N_1_M_0_ | 4.9*2.5 |
