# Supplementary material for: Krüppel‐like factor 4 regulates stemness and mesenchymal properties of colorectal cancer stem cells through the TGF‐β1/Smad/snail pathway
Source: J Cell Mol Med. 2019 Dec 12;24(2):1866–77. doi: 10.1111/jcmm.14882 (PMC6991673; doi:10.1111/jcmm.14882)
Supplement: Supplementary file 3 [file JCMM-24-1866-s003.doc]

**Table S2.** Primers used for qPCR amplification of each mRNA

| GAPDH-F | 5'-GGGGAGCCAAAAGGGTCATCATCT-3' |
| --- | --- |
| GAPDH-R | 5'-GACGCCTGCTTCACCACCTTCTTG-3' |
| KLF4-F | 5'-CGAACCCACACAGGTGAGAA-3' |
| KLF4-R | 5'-TACGGTAGTGCCTGGTCAGTTC-3' |
| CD133-F | 5'-ACCGACTGAGACCCAACATC-3' |
| CD133-R | 5'-GACCGCAGGCTAGTTTTCAC-3' |
| E-cad-F | 5'- GCCCTGCCAATCCCGATGAAA-3' |
| E-cad-R | 5'- GGGGTCAGTATCAGCCGCT-3' |
| ZO-1-F | 5'- TGAGGCAGCTCACATAATGC-3' |
| ZO-1-R | 5'- GGGAGTTGGGGTTCATAGGT-3' |
| Oct4-F | 5'-CTTGCTGCAGAAGTGGGTGGAGGAA-3' |
| Oct4-R | 5'-CTGCAGTGTGGGTTTCGGGCA-3' |
| Sox2-F | 5'-CAAGATGCACAACTCGGAGA-3' |
| Sox2-R | 5'-CATGAGCGTCTTGGTTTTCC-3' |
| Nanog-F | 5'-CAGAAGGCCTCAGCACCTACCTACCCCAGCC-3' |
| Nanog-R | 5'-TCTCTGCAGTCCTGCATGCAGTTCCAGCCAAA-3' |
| TGF-β1-F | 5'- TGGAGCAACATGTGGAACTC-3' |
| TGF-β1-R | 5'- CAGCAGCCGGTTACCAAG-3' |
| N-cad-F | 5'-GGTGGAGGAGAAGAAGACCAG-3' |
| N-cad-R | 5'-GCATCAGGCTCCACAGT-3' |
| CD44-F | 5'-CAACACAAATGGCTGGTACG-3' |
| CD44-R | 5'-GTGTGGTTGAAATGGTGCTG-3' |
| Snail-F | 5'-CCAGACCCACTCAGATGTCAAGAA-3' |
| Snail-R | 5'-GGCAGAGGACACAGAACCAGAAAA-3' |
| Slug-F | 5'-CCCAATGGCCTCTCTCCTCTTT-3' |
| Slug-R | 5'-CATCGCAGTGCAGCTGCTTATGTTT-3' |
| Vim-F | 5'-GCTTCAGAGAGAGGAAGCCGAAAA-3' |
| Vim-R | 5'-CCGTGAGGTCAGGCTTGGAAA-3' |
