# Supplementary material for: Krüppel‐like factor 4 regulates stemness and mesenchymal properties of colorectal cancer stem cells through the TGF‐β1/Smad/snail pathway
Source: J Cell Mol Med. 2019 Dec 12;24(2):1866–77. doi: 10.1111/jcmm.14882 (PMC6991673; doi:10.1111/jcmm.14882)
Supplement: Supplementary file 4 [file JCMM-24-1866-s004.doc]

**Table S3.** The percentages of Lgr5+CD44+EpCAM+ cells in human colorectal cancer cell lines and tissue samples

| Cell lines or tissue samples | Percentage (%) |
| --- | --- |
| DLD-1 | 0.6±0.1 |
| HCT116 | 0.3±0.06 |
| HT29 | 0.25±0.04 |
| patients #1 | 1.1±0.1 |
| patients #2 | 0.5±0.06 |
| patients #3 | 1.5±0.13 |
| patients #4 | 0.9±0.17 |
| patients #5 | 0.2±0.02 |
| patients #6 | 0.9±0.11 |
| patients #7 | 0.3±0.04 |
| patients #8 | 1.4±0.31 |
| patients #9 | 0.2±0.04 |
| patients #10 | 0.32±0.06 |
| patients #11 | 1.12±0.09 |
| patients #12 | 1.3±0.15 |
